# The Immediate Hotspot of Microbial Nitrogen Cycling in an Oil-Seed Rape (*Brassica campestris* L.) Soil System Is the Bulk Soil Rather Than the Rhizosphere after Biofertilization

**DOI:** 10.3390/microorganisms10020247

**Published:** 2022-01-23

**Authors:** Shanghua Wu, Tsing Bohu, Yuzhu Dong, Shijie Wang, Shijie Zhao, Haonan Fan, Xuliang Zhuang

**Affiliations:** 1 Key Laboratory of Environmental Biotechnology, Research Center for Eco-Environmental Sciences, Chinese Academy of Sciences, Beijing 100085, China; shwu@rcees.ac.cn (S.W.); dongyuzhu0929@163.com (Y.D.); sjwang_st@rcees.ac.cn (S.W.); sjzhao_st@rcees.ac.cn (S.Z.); fanhnan@foxmail.com (H.F.); 2 College of Resources and Environment, University of Chinese Academy of Sciences, Beijing 100049, China; 3 State Key Laboratory of Lunar and Planetary Sciences, Macau University of Science and Technology, Taipa, Macau 999078, China; qhu@must.edu.mo; 4 CSIRO Mineral Resources, 26 Dick Perry Avenue, Kensington, WA 6151, Australia; 5 Institute of Tibetan Plateau Research, Chinese Academy of Sciences, Beijing 100101, China

**Keywords:** plant-soil interactions, nitrification inhibitor, competitive and cooperative community, network, *Bacillus amyloliquefaciens*

## Abstract

Biofertilizers are substances that promote plant growth through the efficacy of living microorganisms. The functional microbes comprising biofertilizers are effective mediators in plant-soil systems in the regulation of nitrogen cycling, especially in nitrification repression. However, the deterministic or stochastic distribution of the functional hotspot where microbes are active immediately after biofertilization is rarely investigated. Here, pot experiments with oil-seed rape (*Brassica campestris* L.) were conducted with various chemical and biological fertilizers in order to reveal the distribution of the hotspot after each fertilization. A stimulated dynamic of the nitrogen cycling-related genes in the bulk soil inferred that the bulk soil was likely to be the hotspot where the inoculated bacterial fertilizers dominated the nitrogen cycle. Furthermore, a network analysis showed that bulk soil microbial communities were more cooperative than those in the rhizosphere after biofertilization, suggesting that the microbiome of the bulk soils were more efficient for nutrient cycling. In addition, the relatively abundant ammonia-oxidizing bacteria and archaea present in the networks of bulk soil microbial communities further indicated that the bulk soil was the plausible hotspot after the application of the biofertilizers. Therefore, our research provides a new insight into the explicit practice of plant fertilization and agricultural management, which may improve the implementational efficiency of biofertilization.

## 1. Introduction

Nitrogen is one of the major nutrients limiting plant growth in terrestrial ecosystems, and the nitrogen transformation that is driven by microorganisms in plant-soil systems is one of the most active hotspots of the global nitrogen cycle [1,2]. With an increased use of chemical fertilizers in farmlands, it is important to regulate the nitrogen cycle in plant-soil systems, increase nitrogen usage efficiency, and mitigate nitrogen loss through ammonia volatilization, nitrate leaching, and nitrous oxide emissions. For years, various methods have been used to regulate the transformation of nitrogen in farmland, including those based on abiotic materials, such as biochar, urease inhibitors, and nitrification inhibitors, and biotic methods using soil bacteria and fungi. However, a more environmentally friendly way of regulating the nitrogen cycle in soil is needed.

Plant growth-promoting bacteria (PGPB) are a type of free-living microbe in plant-soil systems that are beneficial to plants. Usually, those microbes are applied in the rhizosphere in order to promote plant growth by phytohormones or to increase N-use efficiency, which play the same role of nitrification inhibitors [3]. However, in our previous work, PGPB, such as *Bacillus amyloliquefaciens* (BA) and *Bacillus subtilis*, have been used in pot and field experiments to mitigate the emission of ammonia and nitrous oxide, and to decrease the leaching of nitrate [3,4,5,6]. These studies have confirmed the potential of biofertilizers in regulating the nitrogen cycle in farmland, one of the best applications was the inhibition of the nitrification process by BA [3]; however, the site of action, whether it is in the rhizosphere or the bulk soil, has not been deciphered to date.

The rhizosphere is a critical zone where roots access water and nutrients, such as nitrogen, and is also believed to be one of the hotspots of microbial reactions because of the complex root exudates and the relationships with the plants [7,8]. Bulk soil is another important zone where plants interact with soil and usually has more diverse bacterial communities compared with that of the rhizosphere, which leads to heterogeneous functions [9]. The difference between the communities residing in these two zones may also show different competitive or cooperative interactions in the community, which would lead to different responses to disturbance, including chemical or microbial fertilizers. Competitive communities are more stable in their response to biotic permutations, such as the inoculation of foreign microbes, when compared to cooperative communities, which are more adaptative to abiotic factors, including nutrients [10]. However, the response and results in the rhizosphere and bulk soils of the two types of fertilizers remain unknown.

Therefore, we hypothesized that (i) bacterial communities in the rhizosphere and bulk soils show different characteristics of competitive and cooperative interactions due to the presence of different nutrients acquired from the root; (ii) the interactions are more competitive in the rhizosphere while more cooperative in the bulk soil; and (iii) the hotspots of the nitrogen cycle, after the addition of chemical fertilizers, are the rhizosphere soils, but this changes to the bulk soil after BA inoculation.

## 2. Materials and Methods

### 2.1. Pot Experiments

A total of 2.5 kg of sieved pre-treated surface soil (0–20 cm, pH < 5.5), collected from a more than 20-year-old upland tea plantation in Changsha (28°34′ N, 113°20′ E), was packed in pots (15 cm in diameter and 20 cm in height). The oil-seed rape cultivar Jingguan (*Brassica campestris* L. cv. Jingguan, Changsha, Hunan, China) was used in this study as a model species because of its wide use in China as a plantation crop. Simply, full seeds were picked up and put into 70% alcohol for 30 s and sterilized in 3% (*v*/*v*) H_2_O_2_ for 10 min followed by a thorough washing with deionized water, before being germinated in a 37 °C thermostat incubator. After 24 h, 10 germinated seeds were selected and transplanted into pots (15.0 cm diameter, 20 cm height).

Three treatments were set up with four replicates each, as follows: inoculation of BA (6 × 10^10^ cells per pot, hereafter referred to as Treatment BN), application of urea (the same total nitrogen was added as Treatment BN, hereafter referred to as Treatment CF), and a control treatment without inoculation of BA or urea (hereafter referred to as Treatment CK). The strain BA EBL11 inoculated in lysogeny broth (LB) media was washed with sterile water three times and then suspended in a concentration of 3.0 × 10^8^ CFU mL^−1^ for inoculation.

The soil moisture content of all treatments was kept consistent throughout the experiment by watering with tap water. Plants were grown in a greenhouse where the day/night temperature were 12/30 °C. After 35-days of incubation, rhizosphere and bulk soils were collected, immediately following frozen-drying for the extraction of DNA.

### 2.2. Soil DNA Extraction and High-Throughput Sequencing

Based on the instructions from the manufacturer, 0.5 g of rhizosphere and bulk soils were prepared and ice-dried for the extraction of total DNA using the FastDNA SPIN kit for soil (MP Biomedicals, Santa Ana, CA, USA).

Then, the bacteria-specific primers 338F and 806R were chosen to amplify the V3-V4 region of the 16S rRNA gene in 25-μL reactions according to previous procedure [3]. Then, the purified PCR products were sequenced in a MiSeq instrument at Central South University, Changsha, China. All raw data have been submitted to the Sequence Read Archive (SRA) database (SAMN06187514-SAMN06187556).

### 2.3. Quantification of Functional Genes in Nitrogen Cycles

Functional genes of both the nitrification and denitrification process (*amoA* of ammonia-oxidizing archaea (AOA), *amoA* of ammonia-oxidizing bacteria (AOB), *nir*K, *nir*S, and *nos*Z) were carried out using Premix Ex Taq (TaKaRa, Japan) and gene-specific primers to quantitative copy number of functional genes in nitrogen cycles for rhizosphere and bulk soil across all treatments. The details referred to Harter et al. [11].

### 2.4. Data Analyses

Based on the Galaxy pipeline at the Research Center for Eco-Environmental Sciences, Chinese Academy of Sciences (http://159.226.240.74:8080/ (accessed on 10 October 2020)), the sequencing data analysis was performed for the classification of all the microorganisms. After the classification of the OTUs, all the potential nitrifiers and denitrifiers were picked out for further analysis. The Shannon index of nitrifiers and denitrifiers in rhizosphere and bulk soils were calculated through PRIMER 7 across all treatments. To elucidate the relationship between species for rhizosphere and bulk soils in different treatments, network analyses were performed on the website of Molecular Ecological Network Analysis (MENA) (http://ieg2.ou.edu/MENA (accessed on 15 October 2020)) and visualized by Gephi (version 0.92, Compiègne, France). All the statistically significant differences (*p* < 0.05) among treatments were determined by one-way ANOVA. The relationship between the two groups of data was evaluated by post-hoc Tukey HSD test to check for statistically significant results.

## 3. Results and Discussion

In order to elucidate the hotspots of the nitrogen cycle after the addition of chemical and biofertilizers, we compared the copy numbers and the alpha diversities of functional species between the rhizosphere and the bulk soils. We found that after the addition of chemical fertilizers, the copy numbers of the five genes, including those involved in both nitrification and denitrification, were much higher than those in bulk soil. The ammonia-oxidizing archaea (AOA) from the bulk soil to the rhizosphere increased from 1.7 × 10^7^ copies/g dry soil to 2.3 × 10^7^, which was an increase of 35%. The ammonia-oxidizing bacteria (AOB) increased from 1.4 × 10^8^ to 1.8 × 10^9^, which was an increase of more than 10 times. The same tendency was observed in the three genes encoding the denitrification process, including *nir*S, *nir*K, and *nos*Z. In addition, the diversity in the rhizosphere was also higher compared with that in the bulk soil. It seems that the hotspots of the CF treatments were the rhizosphere soils.

**Figure 1:**
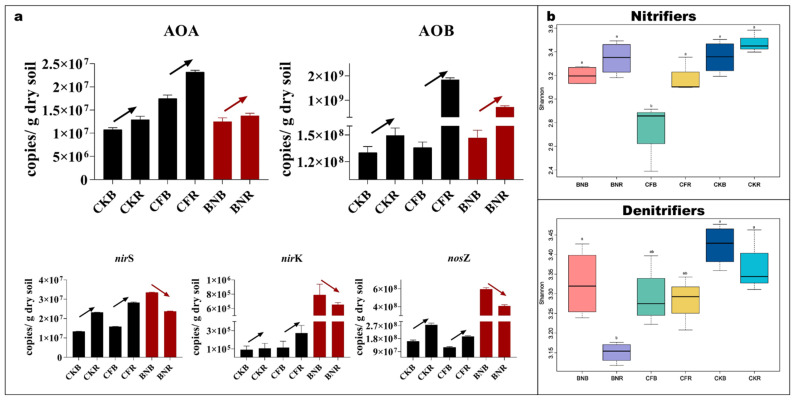
Quantification of functional species involved in nitrification and denitrification processes in rhizosphere and bulk soils. (**a**) Functional gene copies of nitrification (AOA and AOB) and denitrification (*nir*S, *nir*K, and *nos*Z) in rhizosphere and bulk soils; (**b**) the Shannon index of nitrifiers and denitrifiers in the rhizosphere and bulk soils. CKB: Bulk soil in treatments without any fertilizers, CKR: Rhizosphere soil in treatments without any fertilizers, CFB: Bulk soil in treatments with chemical fertilizers, CFR: Rhizosphere soil in treatments with chemical fertilizers, BNB: Bulk soil in treatments with biofertilizers, and BNR: Rhizosphere soil in treatments with biofertilizers.

Nevertheless, after the biofertilizers were applied, the copy numbers and diversities of the nitrification process in the rhizosphere were found to be higher than those in the bulk soil, while the denitrification process was more diverse in the latter (Figure 1). The ammonia-oxidizing archaea (AOA) from the bulk soil to rhizosphere increased slightly from 1.3 × 10^7^ copies/g dry soil to 1.4 × 10^7^. The ammonia-oxidizing bacteria (AOB) increased from 1.5 × 10^8^ to 7.2 × 10^8^. The changes of AOA and AOB indicated that the rhizosphere was the hotspot of the nitrification process. While in the three genes encoding the denitrification process, an opposite tendency was found from the bulk soil to the rhizosphere, all of the three genes’ abundances decreased, which indicate that the bulk soil was the hotspot of denitrification. As found previously, BA could inhibit the nitrification process and enhance the denitrification process [3], suggesting that the hotspots in Treatment BN are the bulk soils, where it decreased the nitrifiers and increased denitrifiers.

This would be a good complement to the regulation and inhibition of nitrification in the plant-soil system, and for the further increase of the nitrogen use efficiency. For most biological nitrification inhibitors, the synthesis and secretion are primarily regulated in the rhizosphere by the availability of nitrogen [12]. However, lots of nitrogen loss was observed in the bulk soil based on the application of the chemical fertilizers, therefore BA would be helpful in the control of these losses.

**Figure 2:**
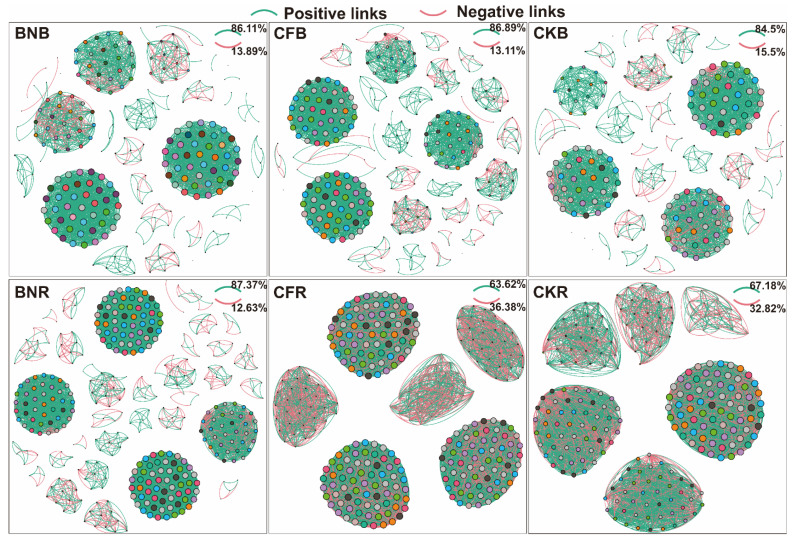
Interaction networks of three different treatments in rhizosphere and bulk soils. CKB: Bulk soil in treatments without any fertilizers, CKR: Rhizosphere soil in treatments without any fertilizers, CFB: Bulk soil in treatments with chemical fertilizers, CFR: Rhizosphere soil in treatments with chemical fertilizers, BNB: Bulk soil in treatments with biofertilizers, and BNR: Rhizosphere soil in treatments with biofertilizers.

In order to find out why the two fertilizer types lead to different nitrogen cycle hotspots; we applied the ecological networks to elucidating the interactions among the residing microbes. We first constructed the networks of the control treatments (Figure 2). In the bulk soil, more negative links were detected than in the rhizosphere, implying that more competitive features are present in the bulk soil than in the rhizosphere [13]. This may be caused by the differences in the nutrients in the two types of communities. In the rhizosphere, nutrients, especially carbon-containing ones, are made readily available by the complex root exudates, allowing the communities to show more features of cooperation [10,14,15]. As mentioned above, competitive communities can better resist species invasion but not nutrient shift; conversely, cooperative communities are susceptible to species invasion but resilient to nutrient change. The bulk soil is made up of highly competitive communities compared with that of the rhizosphere, resulting in different responses to chemical and biofertilizers [10].

Then, based on the same strategies, we constructed another four networks of rhizosphere and bulk soils in the CF and BN treatment groups (Figure 2). It was found that, after inoculation of BA, substantial changes were observed in both the rhizosphere and the bulk soils, despite the minimal change in the nodes included in the networks. The links in the rhizosphere were enhanced while those in the bulk soil diminished, possibly due to the inhibition of nitrification in the bulk soil by BA. The characteristics of cooperation in the communities of both the bulk soil and the rhizosphere was increased, especially in the bulk soil, which indicated a stronger competitive relationship between BA and original species in the bulk soil [13]. These results elucidated the results of Q-PCR, where BA took place more in the bulk soil than in the rhizosphere.

Besides, after the application of BA, there were differences in the response of the functional species in the bulk soil where the hotspots were. It was found that AOA took some of the place of AOB in the modules of the functional groups. Actually, more AOA (nine AOA were detected in BNB while only four were detected in CKB) were linked and found in the four biggest modules, and most of them were positive links between microbes. As for the AOB, fewer AOB (36 AOA were detected in BNB while 44 were detected in CKB) were found in the modules after the inoculation of BA in the treatment BNB. Moreover, more links were detected between AOA and nitrite-oxidizers in the BNB treatment. Usually in farmland, AOB were believed to take on the role of the nitrification process [16], but BA inhibited the activities and links of AOB in the ecological networks, which also supported the results of the bulk soil as the hotspots.

Here, our research investigated the hotspots of PGPB when applied to regulating the nitrogen cycles, and bulk soil was found to be significant in the changing status of the nitrogen cycles. This will give good guidance in the future application of PGPB in crop production. However, despite of the hotspots elucidated in this research, how PGPB could have successfully colonized in the bulk soil and the influence of the indigenous microbes on the effects of PGPB should be studied to a better application.

## 4. Conclusions

We found that *Bacillus amyloliquefaciens* could be a useful nitrification inhibitor and the hotspots of its inhibition take place mainly in the bulk soil, which make it a complement of the rhizosphere to bulk soil inhibition of nitrification. Besides, the rhizosphere and the bulk soil showed different features of competitive and cooperative communities; in the rhizosphere, more competitive patterns were identified. These differences led to the different responses of the functional species of the nitrogen cycle in the rhizosphere and the bulk soils. Our findings reveal that the hotspots of BA regulation are in the bulk soil, which holds major implications in regulating the nitrogen cycle in the oil-seed rape (*Brassica campestris* L.) soil system.

## Data Availability

All raw data have been submitted to the Sequence Read Archive (SRA) database (SAMN06187514-SAMN06187556).
